# Depression in healthcare workers during COVID-19 pandemic: results from Czech arm of HEROES Study

**DOI:** 10.1038/s41598-023-39735-w

**Published:** 2023-08-01

**Authors:** Pavla Cermakova, Barbora Fryčová, David Novák, Marie Kuklová, Katrin Wolfová, Matěj Kučera, Miroslava Janoušková, Jaroslav Pekara, Jana Šeblová, Dominika Seblova

**Affiliations:** 1 Department of Epidemiology, Second Faculty of Medicine, Charles University Prague, V Úvalu 84, 150 06 Prague 5, Czechia; 2 National Institute of Mental Health, Klecany, Czechia; 3 Faculty of Science, Charles University Prague, Prague, Czechia; 4 Faculty of Science, Amsterdam Public Health Research Institute, Vrije Universiteit Amsterdam, Amsterdam, The Netherlands; 5 Third Faculty of Medicine, Charles University Prague, Prague, Czechia; 6 Medical College, Prague, Czechia; 7 Paediatric Emergency Department, Motol University Hospital, Prague, Czechia

**Keywords:** Health occupations, Risk factors, Epidemiology

## Abstract

The pandemic due to COVID-19 brought new risks for depression of health care workers, which may have differently influenced men and women. We aimed to investigate (1) whether health care workers in Czechia experienced an increase in depression during the COVID-19 pandemic, (2) which factors contributed the most to this change, and (3) whether the magnitude of the associations differed by gender. We studied 2564 participants of the Czech arm of the international COVID-19 HEalth caRe wOrkErS (HEROES) Study. Online questionnaire was administered to health care workers in summer 2020 (wave 0) and spring 2021 (wave 1). Depression was defined by reaching 10 or more points on the Patient Health Questionnaire. Logistic regression investigated the association of participant´s characteristics with depression and multivariable decomposition for non-linear models assessed, to what extent the characteristic explained the change in depression occurrence. The prevalence of depression increased twice during the pandemic (11% in wave 0 and 22% in wave 1). Stress accounted for 50% of the difference, experience of death due to COVID-19 for 15% and contact with COVID-19 patients for 14%. Greater resilience and sufficient personal protective equipment were strongly associated with lower occurrence of depression. The protective association of resilience with depression was stronger in men than in women. We conclude that interventions to promote mental health of health care workers in future health crisis should aim at decreasing stress and enhancing resilience. They should be delivered especially to individuals who have contact with the affected patients and may face their death.

## Introduction

The COVID-19 pandemic brought about a challenge for the mental health of healthcare workers (HCWs) due to the high demands of caring for patients, changing work conditions, assignment of new tasks, fear regarding personal safety as well as worries about the health of their families^1^. HCWs have belonged to the most vulnerable population groups during the pandemic, being at a risk for developing depression^2,3^. There are countless studies providing strong evidence of a high burden of depression in HCWs during the pandemic, which were summarized in several systematic reviews and meta-analyses. In a an umbrella review of previously published meta-analyses, the prevalence of depression among HCWs during the COVID-19 pandemic was found to be 25%^2^. Another systematic umbrella review, providing global evidence on almost 170 000 HCWs from 35 countries, indicated that the prevalence of depression ranged from 18 to 36% and was higher among physicians (40%) than among nurses (28%)^3^. Fewer studies tracked the development of depression among HCWs as the pandemic progressed and suggested an increase in depressive symptoms during peaks of the pandemic^4,5^.

There is consensus that services and interventions to enhance mental health need to be implemented to support mental health of HCWs and suggestions were made for risk stratification that could guide intervention delivery^6^. Studies suggest that mental health of HCWs is related to organizational factors, such as workload and contact with patients infected with COVID-19^7^. At the start of the pandemic, HCWs were more interested in occupational protection, social support and getting more rest, however, the available interventions addressed individual psychopathology, suggesting a mismatch between their needs and available services^7^.

Several sociodemographic factors such as gender, profession, age, workplace, work department; and health-related as well as psychological factors including low social support or self-efficacy were found to be associated with increased depressive symptoms^6,8^. Regarding work department, HCWs in the emergency or intensive care departments were found to be particularly likely to perceive an exposure risk^9^. Another survey amongst surgeons found that head and neck specialties had the highest psychological distress^10^. Great differences in mental health exist between various professions working in health care. For example, staff in administration has experienced a particular increase in work pressure during the pandemic, compared to other professions^11^. Another study on psychological differences amongst HCWs found that nurses experienced lower distress when compared to other HCWs^12^. Other authors found strong associations between physical symptoms, in particular headaches and prior chronic illnesses, and poor mental health of HCWs^8,13^.

The a priori greater risks for depression in women became even higher during the pandemic, as women working in health care faced unprecedented challenges at the workplace in combination with increased caring responsibilities due to the closure of care facilities and schools. A scoping review reported that the greater depression risk in women in health care was triggered by a number of individual factors, such as lack of social support or family status, as well as organizational factors, including access to personal protective equipment (PPE), high workload or a lack of recognition at work^14^. It is, therefore, plausible that the risks for the increased depression in HCWs were magnified in women.

Citizens of Czechia, a country situated in Central and Eastern Europe, have greater risk of poor mental health, when compared to their counterparts in Western Europe or Scandinavia^15^. Large treatment gaps for mental disorders, associated stigma, lack of preventive measures and community-based mental health services, frequent alcohol abuse, and unhealthy lifestyle habits might partially explain worse mental health outcomes in Czechia compared to other European countries^16–18^. During the first peak of the pandemic in Czechia, the nationwide state of emergency lasted from 12th March to the 17th of May 2020. Services considered as non-essential were limited and citizens were under stay-at-home orders from the 16th of March^19^. A survey conducted in May 2020 demonstrated that mental health of the Czech population greatly worsened during the pandemic; when compared to November 2017, the prevalence of current affective disorders increased from 7 to 19%, and the prevalence of a current major depressive episode grew from 4 to 12%^20^. The mental health of the population did not improve during the second wave of the pandemic that started in autumn 2020. In November 2020, the prevalence of current affective disorders was estimated to be 21% and the prevalence of a major depressive episode slightly over 12%^21^. Gender differences were apparent, with the prevalence of major depressive episode reaching 14% in women and 10% in men^21^.

Changes in mental health of HCWs during the pandemic have not been systematically studied in Czechia yet. Capitalizing on the Czech longitudinal arm of a large international study, we aim to examine (1) whether HCWs experienced an increase in depression during the COVID-19 pandemic in Czechia, (2) which factors contributed the most to this change, and (3) whether the magnitude of the associations differed by gender. In addition to having a unique sample from a country underrepresented in previous research, another strength of our study is the inclusion of a wide range of staff working in health care, encompassing technical and administrative personnel who faced stressors related to COVID-19 without receiving recognition for their contribution. Further, the present study benefits from a unique design as we can investigate the changes using both repeated cross-sectional surveys as well as a cohort analysis.

## Results

### Analysis of cross-sectional surveys on Sample 1

In wave 0 (n = 1413 individuals, mean age 45 years, 76% women), 11% of the participants had moderate to severe depression. In wave 1 (n = 1620, mean age 46 years, 75% women), 22% of the participants had moderate to severe depression. They differed across the waves in several other characteristics as well (Table 1). In wave 1, there was a higher frequency of individuals with graduate education (61% vs. 54%), physicians (42% vs. 27%) and workers in private sector (20% vs. 14%), when compared to wave 0. The participants in wave 1 also reported more frequently experience of stress (54% vs. 25%), chronic physical illness (31% vs. 23%), contact with COVID-19 patients (69% vs. 17%), patient prioritization (20% vs. 8%), experience of death due to COVID-19 (52% vs. 12%), sufficient PPE (74% vs. 63%) and high trust in the workplace (52% vs. 50%). On the contrary, the participants in wave 1 reported less frequently daily informal caregiving (42% vs. 61%), change in function (37% vs. 40%) and experience of stigmatization, discrimination, or violence (27% vs. 30%) and reached a slightly lower score on the resilience scale (3.3 vs. 3.4), when compared to the participants in wave 0.

**Table 1:** Differences in participant's characteristics between waves in Sample 1.

	Wave 0 (N = 1413)	Wave 1 (N = 1620)
Depression (PHQ > 9); n (%)	154 (10.9%)	363 (22.4%)
Depressive symptoms, median (IQR)	3.0 (1.0, 6.0)	5.0 (2.0, 9.0)
Sociodemographic characteristics		
Age, years, mean ± SD	44.7 ± 11.7	45.6 ± 12.2
Gender: women, n (%)	1079 (76.4%)	1210 (74.7%)
Education, n (%)		
Lower	357 (25.3%)	364 (22.5%)
Undergraduate	296 (20.9%)	272 (16.8%)
Graduate or higher	760 (53.8%)	980 (60.6%)
Occupation, n (%)		
Physician	379 (26.8%)	675 (41.7%)
Nurse or other medical staff	604 (42.7%)	595 (36.7%)
Management	234 (16.6%)	191 (11.8%)
Other	196 (13.9%)	159 (9.81%)
Work sector: private, n (%)	194 (13.7%)	322 (19.9%)
Risk factors for poor mental health		
Daily informal caregiving, n (%)	764 (61.3%)	675 (41.7%)
Stress, n (%)	355 (25.2%)	877 (54.2%)
Chronic physical illness, n (%)	291 (22.9%)	478 (31.1%)
Change in function, n (%)	563 (39.8%)	599 (37.0%)
Contact with COVID-19 patients, n (%)		
Yes	235 (16.6%)	1106 (68.9%)
No	886 (62.7%)	318 (19.8%)
I do not know	292 (20.7%)	182 (11.3%)
Patient prioritization, n (%)		
Yes	88 (8.3%)	319 (20.0%)
No	823 (78.0%)	1049 (65.7%)
Does not apply	144 (13.6%)	229 (14.3%)
Experience of stigmatization, discrimination or violence, n (%)	420 (29.7%)	432 (27.0%)
Experience of death due to COVID-19, n (%)	153 (11.7%)	831 (52.1%)
Protective factors for mental health		
Resilience, mean ± SD	3.4 ± 0.6	3.3 ± 0.7
Sufficient PPE, n (%)	844 (63.4%)	1133 (74.1%)
Trust in workplace, n (%)		
Low	195 (13.8%)	219 (13.7%)
Moderate	516 (36.5%)	552 (34.6%)
High	702 (49.7%)	824 (51.7%)

The data is original, missing data on covariates not imputed.

*PHQ *Patient Health Questionnaire, *PPE* personal protective equipment, *SD* standard deviation, *IQR* interquartile range.

In the multivariable analysis (Table 2), when only sociodemographic characteristics were entered into Model 1, lower age (OR 0.98; 95% CI 0.97–0.99) and being a woman (OR 1.47; 95% CI 1.16–1.88) were associated with higher odds of moderate to severe depression. When risk factors for poor mental health were entered into Model 2, the association with age and gender weakened. In Model 3, when protective factors for mental health were included, lower age and being a woman were no longer associated with moderate to severe depression. In Model 1, physicians had higher odds of moderate to severe depression (OR 1.42; 95% CI 1.03–1.96) when compared to nurses and other medical staff. However, after the risk and protective factors were entered into the model, the association of being a physician to moderate to severe depression became close to unity. On the other hand, individuals who belonged to professions other than medical or managerial had greater odds of moderate to severe depression in both Models 2 and 3 (Model 3: OR 1.58; 95% CI 1.07–2.35).

**Table 2:** Association of participant's characteristics with depression in Sample 1.

	OR (95% CI)		
	Model 1	Model 2	Model 3
Wave 1	**2.38 (1.94–2.93)**	1.24 (0.94–1.63)	1.21 (0.90–1.62)
Sociodemographic characteristics			
Age, years	**0.98 (0.97–0.99)**	**0.99 (0.98–1.00)**	0.99 (0.98–1.00)
Gender: women	**1.47 (1.16–1.88)**	**1.37 (1.05–1.79)**	1.25 (0.94–1.66)
Education			
Lower	Reference		
Undergraduate	0.90 (0.66–1.23)	0.85 (0.61–1.19)	0.88 (0.62–1.25)
Graduate or higher	0.79 (0.58–1.07)	0.90 (0.64–1.25)	0.98 (0.69–1.39)
Occupation			
Physician	**1.42 (1.03–1.96)**	1.06 (0.74–1.52)	0.97 (0.66–1.42)
Nurse or other medical staff	Reference		
Management	1.26 (0.90–1.76)	1.11 (0.77–1.60)	1.44 (0.97–2.12)
Other	1.10 (0.79–1.53)	**1.56 (1.08–2.27)**	**1.58 (1.07–2.35)**
Work sector: private	0.90 (0.68–1.18)	0.98 (0.73–1.33)	0.98 (0.71–1.35)
Risk factors for poor mental health			
Daily informal caregiving		0.97 (0.78–1.20)	1.01 (0.81–1.27)
Stress		**5.59 (4.38–7.15)**	**4.72 (3.66–6.10)**
Chronic physical illness		**1.69 (1.33–2.15)**	**1.48 (1.15–1.90)**
Change in function		1.07 (0.86–1.35)	1.10 (0.86–1.39)
Contact with COVID-19 patients			
Yes		1.23 (0.90–1.68)	1.39 (1.00–1.92)
No	Reference		
I do not know		**1.55 (1.11–2.16)**	1.35 (0.95–1.93)
Patient prioritization			
Yes		**1.57 (1.16–2.12)**	**1.78 (1.29–2.46)**
No	Reference		
Does not apply		0.98 (0.71–1.35)	0.88 (0.63–1.25)
Experience of stigmatization, discrimination or violence		**1.98 (1.59–2.47)**	**1.81 (1.43–2.29)**
Experience of death due to COVID-19		**1.37 (1.04–1.79)**	**1.42 (1.07–1.88)**
Protective factors for mental health			
Resilience			**0.34 (0.28–0.41)**
Sufficient PPE			**0.71 (0.55–0.91)**
Trust in workplace			
Low			**1.42 (1.03–1.95)**
Moderate			Reference
High			1.09 (0.84–1.40)

Missing data on covariates are imputed.

*PPE* personal protective equipment, *OR* odds ratio, *CI* confidence interval.

Significant values are in bold.

In both Models 2 and 3, greater odds of moderate to severe depression were associated with self-reported stress (Model 3: OR 4.72; 95% CI 3.66–6.10), chronic physical illness (Model 3: OR 1.48; 95% CI 1.15–1.90), patient prioritization (Model 3: OR 1.78; 95% CI 1.29–2.46), experience of stigmatization, discrimination or violence (Model 3: OR 1.81; 95% CI 1.43–2.29), and experience of death due to COVID-19 (Model 3: OR 1.42; 95% CI 1.07–1.88). Independent of all sociodemographic characteristics and risk factors for poor mental health (Model 3), greater resilience (OR 0.34; 95% CI 0.28–0.41) and sufficient PPE (OR 0.71; 95% CI 0.55–0.91) were associated with lower odds of moderate to severe depression, while low trust in the workplace was related to its greater odds (OR 1.42; 95% CI 1.03–1.95). In the sensitivity analysis on individuals who had data on depression in both waves, after the exclusion of their observations in wave 1, we found similar results (Supplementary Table S1).

The decomposition for nonlinear models indicated that 79% of the differences in the occurrence of moderate to severe depression across the waves were due to the differences in the baseline characteristics, while 21% were due to the differences in the associations. From the participants´ characteristics that could explain the change in the occurrence of moderate to severe depression, the most salient one was stress, accounting for 50% of the difference. Specifically, if wave 1 levels of stress were equal to wave 0 levels, there would be an expected reduction in the difference in the occurrence of depression corresponding to 50%. Further salient characteristics and the percentage corresponding to the difference in depression across waves were experience of death due to COVID-19% (15%), contact with COVID-19 patients (14%), resilience (9%) and sufficient PPE (5%). Results of the decomposition are presented in Table 3.

**Table 3:** Results of the multivariable decomposition (Sample 1).

	B (95% CI)	%
Sociodemographic characteristics		
Age	−0.00 (−0.00; 0.00)	−0.72
Gender	−0.00 (−0.00; 0.00)	−0.38
Education	0.00 (−0.00; 0.00)	0.51
Occupation	−0.00 (−0.01; 0.00)	−2.04
Work sector	−0.00 (−0.00; 0.00)	−0.48
Risk factors for poor mental health		
Daily informal caregiving	−0.00 (−0.01; 0.00)	−2.37
Stress	**0.06 (0.04; 0.07)**	**50.12**
Chronic physical illness	**0.00 (0.00; 0.01)**	**3.20**
Change in function	−**0.00 (**−**0.00; **−**0.00)**	−**0.97**
Contact with COVID-19 patients	**0.02 (0.01; 0.02)**	**13.58**
Patient prioritization	0.00 (−0.00; 0.00)	1.57
Experience of stigmatization, discrimination or violence	−**0.00 (**−**0.00; **−**0.00)**	−**1.55**
Experience of death due to COVID-19	**0.02 (0.00; 0.03)**	**14.48**
Protective factors for mental health		
Resilience	**0.01 (0.01; 0.01)**	**9.07**
Sufficient PPE	−**0.01 (**−**0.01; **−**0.00)**	−**4.82**
Trust in workplace	−0.00 (−0.00; 0.00)	−0.38

*PPE* personal protective equipment, *CI* confidence interval.

Positive percentage means that if wave 1 levels of the characteristic were equal to wave 0 levels, there would be expected reduction in the difference in prevalence of depression corresponding to that amount of percentage. Negative percentage means that wave 1 levels of the characteristic were equal to wave 0 levels, there would be expected increase in the difference in prevalence of depression corresponding to that amount of percentage. For the multivariable decomposition, we used 3 datasets to compare the results across the models for consistency: complete case sample, the 1st imputed and the 10th imputed dataset. As the differences were minimal, we present in this table results from the 1st imputed dataset.

Significant values are in bold.

From the five most salient characteristics, four of them had an unequal distribution between genders (Supplementary Table S2): stress was more often reported by women (women 42% vs. men 35%). On the contrary, men reported more frequently having contact with COVID-19 patients (men 52% vs. women 42%), experience of death due to COVID-19 (men 39% vs. women 30%). Men had also a higher resilience score than women (3.5 vs. 3.3). Men and women did not differ in the distribution of sufficient PPE. Next, we tested if gender was an effect modifier in the association of these four characteristics with depression and detected only an interaction between gender and resilience (OR 1.85; 95% CI 1.18–2.92; p = 0.008). Stratification by gender showed that the magnitude of the effect of resilience was greater among men (OR 0.16; 95% CI 0.10–0.26) than among women (OR 0.39; 95% CI 0.32–0.47).

### Longitudinal cohort analysis on Sample 2

Table 4 shows that except for sufficient PPE, the four most salient characteristics that were found to explain the increase in moderate to severe depression on Sample 1 were also associated with increased odds of depression in Sample 2. Specifically, participants who reported stress had more than four times the odds of depression (OR 4.39; 95% CI 2.23–8.64). Participants who had contact with patients with COVID-19 (OR 3.42; 95% CI 1.45–8.06) as well as those who did not know if they had contact with such patients (OR 2.76; 95% CI 1.06–7.17) and individuals who had experience with death due to COVID-19 (OR 2.04; 95% CI 1.03–4.06) had increased odds of depression. On the contrary, greater resilience was associated with lower odds of depression (OR 0.33; 95% CI 0.20–0.54). There were no interactions with gender.

**Table 4:** Associations of participants’ characteristics with depression in Sample 2.

	OR (95% CI)
Age	1.01 (0.99–1.04)
Gender: women	0.81 (0.41–1.63)
Stress	**4.39 (2.23–8.64)**
Contact with COVID-19 patients	
Yes	**3.42 (1.45–8.06)**
No	Reference
I do not know	**2.76 (1.06–7.17)**
Experience of death due to COVID-19	**2.04 (1.03–4.06)**
Resilience	**0.33 (0.20–0.54)**
Sufficient PPE	0.74 (0.38–1.41)

Missing data on covariates are imputed.

*PPE* personal protective equipment, *OR* odds ratio, *CI* confidence interval.

Significant values are in bold.

## Discussion

Capitalizing on the Czech arm of the international HEROES project, we found that the occurrence of moderate to severe depression in HCWs increased twice between the end of the first peak and the height of the second peak of the COVID-19 pandemic. This change was explained the most by increased stress, contact with COVID-19 patients, and experience of death due to COVID-19. Greater resilience and, to a smaller extent, sufficient PPE were found as protective factors against moderate to severe depression. We did not find that the risk factors had a greater influence on women than on men. Interestingly, resilience seems to be a stronger protective factor against depression for men than for women.

The finding of a great increase in the burden of depression among HCWs is in line with research on community-dwelling individuals showing a rise in depression, anxiety, and stress between the first and the second wave of the pandemic^22^. We found that greater perceived stress accounted for about 50% of the increased occurrence of depression during the pandemic. Perceived stress has been consistently found to be a risk factor for depression in HCWs over the course of COVID-19 pandemic. For example, in a cross-sectional study of 588 health care workers in China, from February to March 2020, self-perceived stress was positively associated with depression as well as anxiety^23^. In an one-year prospective study of 120 anesthetists working in the COVID-19 department in Rome, self-reported stress was associated with increasing prevalence of depression^24^. In this study, stress has been from 39% explained by the isolation at work and a reduced perception of organizational justice. The role of stress in depression has been also supported by several theoretical and animal models^25^. Regarding the explanation of this phenomenon in the pandemic on the populational level, Wong et. al confirmed the impact of repeated exposure to social-unrest-related traumatic events, pandemic-related events, and personal stressful life events^26^.

Other strong risk factors for moderate to severe depression of HCWs were contact with COVID-19 patients and experience of death due to COVID-19. These exposures may have influenced the occurrence of depression through other ways, which were not considered in our study. For example, anxiety, psychological or physical exhaustion or moral distress experienced when facing the coexistence of countless deaths, long work shifts with the most diverse unknowns and demands in the treatment of COVID-19 patients may have all contributed to depression. HCWs were reaching their limits, especially when dealing with older patients with death prospects, knowing that HCWs, not a relative, are inevitably the last people a dying COVID-19 patient will see^27^.

On the contrary, greater resilience and somewhat also sufficient PPE were found as protective mechanisms against moderate to severe depression. A rapid review published at the beginning of the COVID-19 pandemic^28^ showed that resilience is a protective factor for general mental health problems (including depression) among HCWs. However, the same study did not find out that confidence in equipment and infection control measures would be a determinant for general mental health. Such findings about the protective role of PPE on depression were published afterwards during the COVID-19 pandemic^29–31^. A study on community-dwelling individuals from two countries with contrasting approaches towards wearing face masks in public also found that the use of face mask at the community level may be related to better mental health^32^.

Even though depression was more common among women than men, gender was not independently associated with greater odds of moderate to severe depression, when all risk and protective factors were accounted for. Further, our study does not suggest that some pandemic-related risk factors, such as informal caregiving, had a stronger influence on the occurrence of moderate to severe depression in women compared to men. Women have higher burden of depression irrespective of the pandemic, therefore, the disproportionately greater burden of depression in women might not necessarily be a result of pandemic-related factors. Our results are in contrast with previous studies on the general population that reported increased risk of depression among women due to the pandemic^33,34^. In general, women were more likely to perceive the pandemic as a serious health risk, were more willing to comply with restrictive measures^35^, and, as a result, experienced greater fear^33^. It is possible that attitudes and behaviours among health care professionals vary less by gender than in general population due to the nature of the occupation.

However, resilience was found to have a greater protective effect against moderate to severe depression in men than in women. Resilience is a capacity to cope with stress and higher resilience is associated with positive and adaptive coping strategies^36^. Studies also suggest that high levels of resilience and positive coping strategies could enhance personal growth, which is a desirable outcome of traumatic events^37^, and that greater resilience is associated with fewer symptoms of post-traumatic stress disorder among HCWs^38^. A previous study identified that men and women differ in their coping strategies^39^. The mechanism explaining the gender difference found in our study is not clear and many genetic as well as environmental factors may play a role, possibly also the conjunction of both work and family care related stressors. We suggest that the already higher baseline resilience in men may have enabled them to utilize more adaptive coping strategies, which further decreased their depressive symptoms.

Even though their weight in explaining the increased prevalence of depression during the pandemic was small, we identified some other characteristics associated with moderate to severe depression. The type of occupation seems to matter in relation to the risk of depression. In the unadjusted analysis, in accord with a previous study^3^, physicians had a greater chance of depression, when compared to nurses. However, this association was explained when risk factors for poor mental health were accounted for. Interestingly, even after adjustment for risk and protective factors, participants whose profession was other than physicians, nurses, or managers, had greater odds of depression. In this category could for example be supporting staff like administrative workers, IT technicians, cleaning staff or technical personnel. Their workload also increased, and they were assigned new tasks and faced new stressors, without any previous training, but their efforts remained invisible. Nurses, physicians, and other medical personnel were applauded and publicly celebrated. Later the financial benefits for wellness were addressed only to medical staff. The non-medical staff sometimes expressed disappointment and feeling of neglection in open parts of the questionnaire. We, therefore, suggest that this neglection may have contributed to their higher risk of moderate to severe depression.

In addition, chronic physical illness, patient prioritization, experience of stigmatization, discrimination, or violence, and low trust in workplace was also associated with greater odds of depression, which is in accord with previous studies^40–44^. COVID-19 pandemic brought about the need to prioritize patients, which causes moral dilemmas and ethically inappropriate actions. Such moral distress also contributes to depression^45^.

This study has several limitations. First, there is a sampling bias as the participants were not selected randomly and are, therefore, not entirely representative of all HCWs in Czechia. Second, non-response bias, which may be associated with mental health, could possibly influence our study, but in which direction is not clear. Third, the relatively low sample size prevented us from accounting for a wider range of characteristics in the longitudinal analysis. In spite of these limitations, we propose that in case of the future health crisis like the COVID-19 pandemic, health care systems need to be better prepared to mitigate the risks for mental health of HCWs. Interventions need to be delivered providing techniques on how to decrease stress and increase resilience. To maximize accessibility, interventions could be also delivered digitally. For example, online cognitive behavioural therapy has been found effective in improving mental health in the general population^46,47^. Such interventions should be targeted especially at individuals who have contact with the affected patients and may face their death. However, occupations other than physicians, nurses, and managers, who work within the health care systems, should not be omitted from such programs and more needs to be known about their needs and experiences during health crises.

## Methods

### Participants

We studied participants enrolled in the Czech arm of the international COVID-19 HEalth caRe wOrkErS (HEROES) Study. HEROES is a global prospective cohort study with the aim to evaluate the impact of COVID-19 pandemic on mental health of HCWs in 26 countries across 4 continents, which was previously described in detail^1^. Data on demographics, mental health, support needs, and provision were collected via an online questionnaire. The data collection in Czechia was initiated in June 2020 (wave 0: 24th of June to 30th of August) after the drop of the first peak of the pandemic. A follow-up survey was conducted in spring 2021 (wave 1: 15th of February to 31st of April) at the height of the second peak of the pandemic. Eligible respondents were workers in healthcare or social services, including physicians, nurses, paramedics, or social workers. Also non-clinical, technical and administrative personnel were invited to participate as they had also new tasks during the pandemic. The respondents were invited to participate through hospital administrators, scientific societies, professional bodies, and associations. In wave 0, 1778 individuals participated (76% women; 44% nurses and 28% physicians), and 1840 individuals took part in wave 1 (75% women; 37% nurses and 42% physicians). From them, 623 individuals (35% of respondents in wave 0) participated in both waves.

All participants gave informed consent prior to survey completion. The HEROES Study was approved by the Columbia University Institutional Review Board. The Czech arm of the HEROES Study was approved by the Ethics Committee of the Ministry of Health as well as the Ethical Review Board of the University Hospital Motol, Prague, Czechia. All methods were performed in accordance with relevant guidelines and regulations.

### Depression

Information on moderate to severe depression was derived from the Patient Health Questionnaire (PHQ-9). PHQ-9 is a nine-item diagnostic instrument that measures the severity of depression, is sensitive to change, has high internal consistency (α = 0.90), test–retest reliability (r = 0.74), and is widely used and validated globally including in Czechia^48–50^. Each of the nine items corresponds to one of the *DSM-IV* Diagnostic Criterion A symptoms for major depressive disorder (decreased interest, feeling down, sleeping problems, little energy, changes in appetite, decreased confidence, trouble concentrating, changed pace, suicidal thoughts). Participants are asked how often, during the last 2 weeks, they have been bothered by each of the depressive symptoms. Response options are “not at all”, “several days”, “more than half the days”, and “nearly every day”, scored as 0, 1, 2 and 3, respectively. The PHQ-9 scores range from 0 to 27. A cut-off point between 8 and 11 points is recommended to capture major depression according to meta-analyses of validation studies^51,52^. The scores of ≥ 5,  ≥ 10 and ≥ 15 were suggested to represent mild, moderate and severe levels of depression^53^. In the present study, we chose the cut-off of point of ≥ 10 points to capture moderate or severe depression.

### Other characteristics

Participants’ characteristics were chosen based on previous literature^54–62^ as factors associated with depressive symptoms in HCWs during the COVID-19 pandemic. They were divided into three groups: sociodemographic characteristics, risk factors for poor mental health, and protective factors for mental health. Self-reported sociodemographic characteristics were age (years), gender (men/women), education (lower [primary, secondary, or technical – professional training]/undergraduate/graduate or higher), occupation (physician/nurse or other medical staff/management / other) and work sector (public/private).

Risk factors for poor mental health included daily informal caregiving (living with or having someone under care – individuals under 18 years of age or over 65 years of age, or individuals with disability; yes/no), stress (feeling constantly under stress/strain during the past week; yes/no; while yes stands for responses “somewhat more than usual” or “much more than usual” and no for “no, not at all or no more than usual”), chronic physical illness (presence of a chronic physical illness before the pandemic; yes/no), change in function (assignment to a new team or assignment of new functions since the beginning of the pandemic; yes/no), contact with COVID-19 patients (close contact with suspect or confirmed COVID-19 patient within the last seven days; yes/no/I don’t know), patient prioritization (having had to decide how to prioritize patients with COVID-19; yes/no/does not apply), experience of stigmatization, discrimination, or violence (having felt stigmatized or discriminated against or having experienced violence as a HCW due to the COVID-19 pandemic; yes/no) and experience of death due to COVID-19 (close contact at work with someone or caring for a patient who later passed away; yes/no).

Protective factors for mental health were resilience (assessed by the Brief Resilience Scale^63^), sufficient PPE (yes/no) and trust in workplace (trusting that the workplace can manage the COVID-19 pandemic; low/moderate/high). In some instances, the phrasing of the questions or their response categories differed in wave 0 and wave 1, therefore, we merged and modified the categories to ensure comparisons between the waves, reduce the number of categories due to their imbalance across waves or improve model convergence (see the code in Supplement).

### Statistical analysis

**Figure 1:**
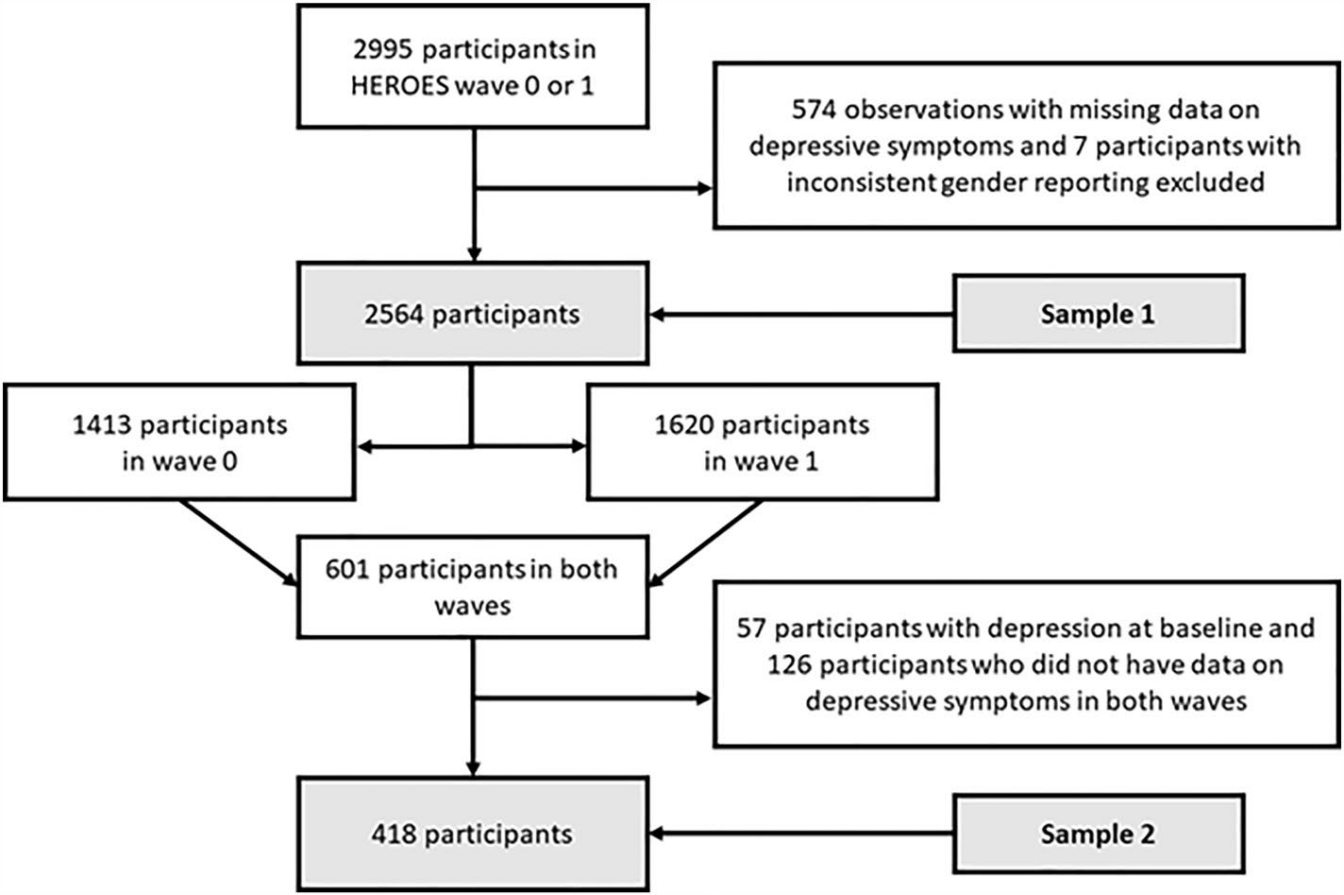
Selection of the study participants.

We performed the statistical analysis in two steps using two analytical samples (Sample 1 and Sample 2). First, from all 2995 participants in wave 0 or wave 1, we excluded those who had missing data on depressive symptoms in one of the waves and those who reported their gender inconsistently across waves or reported their gender other than man or woman, leaving 2564 individuals in Sample 1. Next, from the 601 individuals that took part in both waves, we excluded those who did not have data on depressive symptoms in both waves and those who had depression at baseline, leaving 418 participants in Sample 2. Selection of the samples is presented in Fig. 1. We used the Multivariate Imputation by Chained Equation (MICE) in R^64^ to impute the missing data on characteristics other than depression and gender (10 imputations).

Sample 1 was used for the *analysis of cross-sectional surveys* to investigate whether the occurrence of depression differed across waves, and which factors were associated with the change. In descriptive analysis, we present the characteristics of participants as frequency (*n*, %), mean ± standard deviation (SD) or median and interquartile range (IQR). In multivariable analysis, we used binary logistic regression to estimate odds ratio (OR) with a 95% confidence interval (CI) for the associations of participants´ characteristics with depression. Model 1 includes wave (wave 1 vs. wave 0) and sociodemographic characteristics, Model 2 also adds risk factors for poor mental health, and Model 3 additionally also adds protective factors for mental health. In sensitivity analysis, individuals who had data on depression in both waves were excluded from wave 1, thus, each participant contributed with only one observation.

In order to investigate to what extent the participants´ characteristics explain the differences in the prevalence of depression between wave 0 and 1, we used decomposition for nonlinear models^65^. The decomposition examines if the differences in the occurrence of depression between wave 0 and wave 1 are mainly due to the differences in the exposures across the waves or due to the differential association between the predictors and depression across waves. Next, we assessed effect modification by gender, by performing tests of statistical interaction (participant´s characteristic x gender to predict depression). The characteristics were selected if they had an unequal distribution between men and women in descriptive analysis on Sample 1 and they were among the most salient factors that explained more than 5% of the difference in the occurrence of depression between waves, based on the results from the decomposition. We tested the interaction separately for each characteristic in the fully adjusted Model 3. Likelihood ratio (LR) test comparing the nested models with and without interaction was used to assess the interaction effect.

Sample 2 was used for a *longitudinal cohort analysis* to investigate whether the occurrence of depression changed in the individuals who took part in both waves and which factors were associated with it. In this analysis on a smaller sample, which does not allow for a precise estimation of associations of all characteristics, we aimed to replicate the results of the previous step and entered participants´ characteristics, which were found to be the most salient factors explaining the change in the occurrence of depression between waves based on the decomposition in the *analysis of cross-sectional surveys* on Sample 1, adjusting for age and gender. In the end, we tested effect modification by gender for the association of the participants´ characteristics with depression. The analyses were performed in R version 4.1.2, except for the decomposition, which was conducted using the command mvdcmp in Stata/IC, version 15.1.

## Supplementary Information

Supplementary Information.

## Data Availability

Data is available from the corresponding author upon reasonable request.
