# Supplementary material for: Depression in healthcare workers during COVID-19 pandemic: results from Czech arm of HEROES Study
Source: Sci Rep. 2023 Aug 1;13:12430. doi: 10.1038/s41598-023-39735-w (PMC10394070; doi:10.1038/s41598-023-39735-w)

**SUPPLEMENT**

**Supplementary Table S1** Sensitivity analysis: Association of participants´ characteristics with depression in Sample 1, after the exclusion of the observations in wave 1 in individuals who took part in both waves

|  | OR (95% CI) | | |
| --- | --- | --- | --- |
|  | Model 1 | Model 2 | Model 3 |
| **Wave 1** | **2.48 (1.99-3.10)** | 1.28 (0.94-1.74) | 1.25 (0.90-1.74) |
| **Sociodemographic characteristics** |  |  |  |
| Age, years | **0.98 (0.97-0.99)** | **0.99 (0.97-1.00)** | 0.99 (0.98-1.00) |
| Gender: women | **1.51 (1.15-1.97)** | **1.39 (1.04-1.87)** | 1.27 (0.93-1.74) |
| Education |  |  |  |
| Lower |  | | |
| Undergraduate | 0.88 (0.62-1.23) | 0.83 (0.57-1.20) | 0.87 (0.59-1.28) |
| Postgraduate | 0.73 (0.52-1.03) | 0.84 (0.58-1.22) | 0.89 (0.60-1.32) |
| Occupation |  |  |  |
| Physician | **1.53 (1.07-2.21)** | 1.15 (0.77-1.72) | 1.11 (0.73-1.70) |
| Nurse or other medical staff |  | | |
| Management | 1.23 (0.84-1.80) | 1.13 (0.75-1.72) | **1.64 (1.05-2.56)** |
| Other | 1.10 (0.77-1.59) | 1.46 (0.97-2.18) | **1.56 (1.01-2.39)** |
| Work sector: private | 0.93 (0.69-1.27) | 0.98 (0.70-1.36) | 0.97 (0.68-1.38) |
| **Risk factors for poor mental health** |  |  |  |
| Daily informal caregiving |  | 0.95 (0.74-1.20) | 0.99 (0.77-1.27) |
| Stress |  | **5.83 (4.45-7.63)** | **4.99 (3.76-6.61)** |
| Chronic physical illness |  | **1.61 (1.23-2.10)** | **1.38 (1.04-1.83)** |
| Change in function |  | 1.05 (0.82-1.35) | 1.04 (0.80-1.35) |
| Contact with COVID-19 patients |  |  |  |
| Yes |  | 1.09 (0.77-1.55) | 1.21 (0.83-1.76) |
| No |  | | |
| I do not know |  | 1.32 (0.92-1.91) | 1.15 (0.78-1.70) |
| Patient prioritization |  |  |  |
| Yes |  | **1.47 (1.04-2.06)** | **1.62 (1.13-2.33)** |
| No |  | | |
| Does not apply |  | 1.03 (0.71-1.49) | 0.90 (0.61-1.34) |
| Experience of stigmatization,  discrimination or violence |  | **2.08 (1.63-2.65)** | **1.93 (1.49-2.50)** |
| Experience of death due to COVID-19 |  | **1.43 (1.06-1.94)** | **1.48 (1.08-2.03)** |
| **Protective factors for mental health** |  |  |  |
| Resilience |  |  | **0.33 (0.27-0.41)** |
| Sufficient PPE |  |  | **0.73 (0.55-0.96)** |
| Trust in workplace |  |  |  |
| Low |  |  | **1.46 (1.03-2.06)** |
| Moderate |  |  |  |
| High |  |  | 1.07 (0.80-1.42) |

*Note : PPE – personal protective equipment; OR- odds ratio; CI – confidence interval. Missing data on covariates are imputed.*

**Supplementary Table S2** Differences in participants´ characteristics between men and women in Sample 1

|  | Men  (N=744) | Women  (N=2289) | P value |
| --- | --- | --- | --- |
| Depression (PHQ >9); n (%) | 103 (13.8%) | 414 (18.1%) | 0.009 |
| Depressive symptoms, median (IQR) | 3.00 (1.00, 7.00) | 4.00 (2.00, 8.00) | <0.001 |
| **Sociodemographic characteristics** |  |  |  |
| Age, years, mean ± SD | 44.8 ± 13.4 | 45.3 ± 11.4 | 0.392 |
| Education, n (%) |  |  |  |
| Lower | 100 (13.4%) | 621 (27.1%) | <0.001 |
| Undergraduate | 103 (13.8%) | 465 (20.3%) |  |
| Postgraduate | 540 (72.6%) | 1200 (52.4%) |  |
| Occupation, n (%) |  |  |  |
| Physician | 365 (49.1%) | 689 (30.1%) | <0.001 |
| Nurse or other medical staff | 198 (26.6%) | 1001 (43.7%) |  |
| Management | 110 (14.8%) | 315 (13.8%) |  |
| Other | 71 (9.5%) | 284 (12.4%) |  |
| Work sector: private, n (%) | 106 (14.2%) | 410 (17.9%) | 0.025 |
| **Risk factors for poor mental health** |  |  |  |
| Daily informal caregiving, n (%) | 359 (48.3%) | 1080 (47.2%) | 0.608 |
| Stress, n (%) | 262 (35.2%) | 970 (42.4%) | <0.001 |
| Chronic physical illness, n (%) | 206 (27.7%) | 563 (24.6%) | 0.126 |
| Change in function, n (%) | 281 (37.8%) | 881 (38.5%) | 0.765 |
| Contact with COVID-19 patients, n (%) |  |  |  |
| Yes | 384 (51.6%) | 957 (41.8%) | <0.001 |
| No | 259 (34.8%) | 945 (41.3%) |  |
| I do not know | 97 (13.0%) | 377 (16.5%) |  |
| Patient prioritization, n (%) |  |  |  |
| Yes | 145 (19.5%) | 262 (11.4%) | <0.001 |
| No | 439 (59.0%) | 1433 (62.6%) |  |
| Does not apply | 71 (9.5%) | 302 (13.2%) |  |
| Experience of stigmatization, discrimination or violence, n (%) | 185 (24.9%) | 667 (29.1%) | 0.027 |
| Experience of death due to COVID-19, n (%) | 293 (39.4%) | 691 (30.2%) | <0.001 |
| **Protective factors for mental health** |  |  |  |
| Resilience, mean ± SD | 3.50 ± 0.66 | 3.31 ± 0.650 | <0.001 |
| Sufficient PPE, n (%) | 505 (67.9%) | 1472 (64.3%) | 0.118 |
| Trust in workplace, n (%) |  |  |  |
| Low | 100 (13.4%) | 314 (13.7%) | <0.001 |
| Moderate | 215 (28.9%) | 853 (37.3%) |  |
| High | 424 (57.0%) | 1102 (48.1%) |  |

*Note : PHQ - Patient Health Questionnaire; PPE – personal protective equipment; SD – standard deviation; IQR – interquartile range. The p values come from independent samples t-test (age, resilience), Mann-Whitney test (depressive symptoms) or  χ^2^ test (other variables). The data is original, missing data not imputed.*

**Supplementary Table S3** Differences in participants´ selected characteristics between waves in Sample 2

|  | Wave 0 | Wave 1 |
| --- | --- | --- |
| Depression (PHQ >9); n (%) | 0 (0%) | 55 (13.2%) |
| Age, years, mean ± SD | 46.1 (11.9) | 47.1 (11.9) |
| Gender: women, n (%) | 315 (75.4%) | 315 (75.4%) |
| Stress, n (%) | 87 (20.8%) | 202 (48.4%) |
| Contact with COVID-19 patients, n (%) |  |  |
| Yes | 64 (15.3%) | 242 (58.2%) |
| No | 263 (62.9%) | 116 (27.9%) |
| I do not know | 91 (21.8%) | 58 (13.9%) |
| Experience of death due to COVID-19, n (%) | 35 (8.95%) | 189 (46.0%) |
| Resilience, mean ± SD | 3.47 (0.605) | 3.39 (0.648) |
| Sufficient PPE, n (%) | 263 (66.1%) | 302 (76.5%) |

*Note : PHQ - Patient Health Questionnaire; PPE – personal protective equipment; SD – standard deviation; IQR – interquartile range. The data is original, missing data not imputed.*

**CODE**

# Selecting ID of participants with inconsistent gender or other gender than Male/Female

inconsistent_gender <- df1 %>%

filter((genderW0 == 'Other gender') | (genderW0 != genderW1)) %>%

pull(id)

# Necessary modifications prior to imputation due to wave comparison or to reduce the number of categories where appropriate

df_mice <- df1 %>% mutate(

# Caregiving

scdm_sd08new = ifelse((scdm_sd08 == 1 | scdm_sd09 == 1 | scdm_sd10 == 1), 1, 0),

# Prioritization

prior_W0 = case_when(ep_ep32 == 3 ~ NA_integer_, TRUE ~ as.integer(ep_ep32)),

PPE_mice_W0 = case_when(mp_mp19 == 4 ~ NA_integer_, TRUE ~ as.integer(mp_mp19)),

# Sufficient PPE

PPE_mice_W1 = case_when(mpv2_mp19 == 4 ~ NA_integer_, TRUE ~ as.integer(mpv2_mp19)),

#Physical illness

phys_illW0 = case_when(sp_sp64 == 2 ~ NA_integer_,TRUE ~ as.integer(sp_sp64)),

#Experience of death

mp_mice_mp25a = case_when(mp_mp25a == 2 ~ NA_integer_, TRUE ~ as.integer(mp_mp25a)),

mp_mice_mp25b = case_when(mp_mp25b == 2 | mp_mp25b == 3 ~ NA_integer_, TRUE ~ mp_mp25b),

mpv2_mice_mp25b = case_when(mpv2_mp25b == 2 | mpv2_mp25b == 3 ~ NA_integer_, TRUE ~ mpv2_mp25b)

) %>% rename(

phys_illW1 = spv2_sp64,

prior_W1 = mpv2_ep32,

sector_W0 = tr_tr11,

sector_W1 = trv2_tr11,

redeployment_W0 = tr_tr15,

redeployment_W1 = trv2_tr15,

patient_contact_W0 = mp_mp18,

patient_contact_W1 = mpv2_mp18,

care_giving_W0 = scdm_sd08new,

care_giving_W1 = scdmv2_sd08new,

mpv2_mice_mp25aa = mpv2_mp25aa

) %>% filter( # remove respondents with inconsistent gender between waves from main analysis or other gender than Male/Female

(!id %in% inconsistent_gender)

)

# Mice imputation on raw data produces data frame named long

# Modifications of variables after imputation

long_modif <- long %>%

mutate(

# Sufficient PPE

PPE = case_when(

PPE == 0 | PPE == 1 | PPE == 2 ~ 0,

PPE == 3 ~ 1),

# Calculation of PHQ score

PHQ_tot =

phq_phq68 + phq_phq69 + phq_phq70 + phq_phq71 + phq_phq72 +

phq_phq73 + phq_phq74 + phq_phq75 + phq_phq76,

# Threshold for depression: PHQ score greater than 9

depression = ifelse(PHQ_tot > 9, 1, 0),

# Calculation of BRS score (reverse coding)

re_re58_rev = 6 - re_re58,

re_re60_rev = 6 - re_re60,

re_re63_rev = 6 - re_re63,

brs_questions =

ifelse(is.na(re_re57), 0, 1)

+ ifelse(is.na(re_re58_rev), 0, 1)

+ ifelse(is.na(re_re59), 0, 1)

+ ifelse(is.na(re_re60_rev), 0, 1)

+ ifelse(is.na(re_re62), 0, 1)

+ ifelse(is.na(re_re63_rev), 0, 1),

brs_score = ifelse(

brs_questions == 6,

(ifelse(is.na(re_re57), 0, re_re57)

+ ifelse(is.na(re_re58_rev), 0, re_re58_rev)

+ ifelse(is.na(re_re59), 0, re_re59)

+ ifelse(is.na(re_re60_rev), 0, re_re60_rev)

+ ifelse(is.na(re_re62), 0, re_re62)

+ ifelse(is.na(re_re63_rev), 0, re_re63_rev))

/ brs_questions, NA),

# Expirience of death

exp_death = case_when(

(mp_mice_mp25a == 1 | mp_mice_mp25b == 1) ~ 1,

(mp_mice_mp25a == 0 | mp_mice_mp25b == 0) ~ 0),

# Trust in workplace

trust_workplace = case_when(

mp_mp25f == 0 | mp_mp25f == 1 ~ 0,

mp_mp25f == 2 ~ 1,

mp_mp25f == 3 | mp_mp25f == 4 ~ 2

),

# Stress

stress_level = ifelse(ghq_ad35 == 0 | ghq_ad35 == 1, 0,ifelse(ghq_ad35 == 2 | ghq_ad35 == 3, 1, ghq_ad35)),

# Expirience of discrimination, stigmatization and/or violence

discrimination_violence = case_when(

mp_mp25c == 3 | mp_mp25c == 4| mp_mp25e == 3 | mp_mp25e == 4 ~ 1,

mp_mp25c == 1 | mp_mp25c == 2 | mp_mp25e == 1 | mp_mp25e == 2 ~ 0)

)

Visualization of changes in categorical variables in Sample 2

Visualizations on non-imputed data, category missing excluded.

Stress

df3 %>%
 filter(!is.na(stress_level)) %>%
 ggplot(aes(x = wave, fill = stress_level)) +
 geom_bar(position = "dodge") + labs(fill = "Stress", x = "Wave", y = "Count", tag="A") +
 scale_fill_brewer(palette = "Paired") +
 theme(legend.position="top")


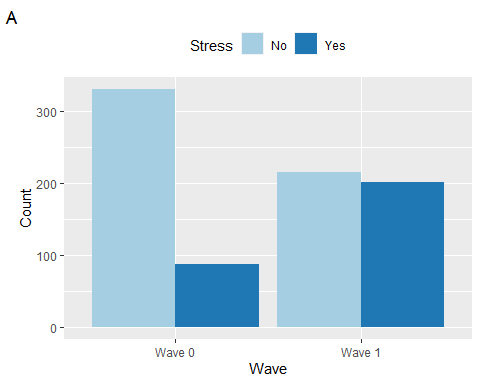


Contact with COVID-19 patients

df3 %>%
 filter(!is.na(patient_contact)) %>%
 ggplot(aes(x = wave, fill=patient_contact)) +
 geom_bar(position = "dodge") + labs(fill = "Contact with Covid-19 patients", x = "Wave", y = "Count", tag="B") +
 scale_fill_brewer(palette = "Paired") +
 theme(legend.position="top")


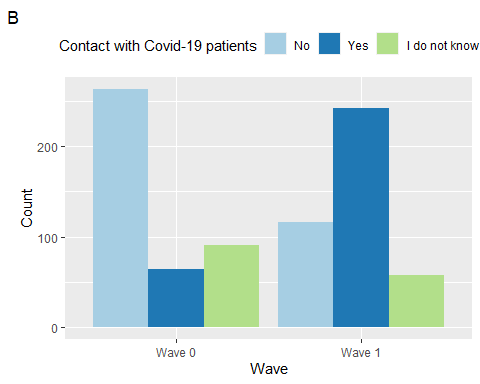


Experience of death due to COVID-19

df3 %>%
 filter(!is.na(exp_death)) %>%
 ggplot(aes(x = wave, fill = exp_death)) +
 geom_bar(position = "dodge") + labs(fill = "Experience of death", x = "Wave", y = "Count", tag="C") +
 scale_fill_brewer(palette = "Paired") +
 theme(legend.position="top")


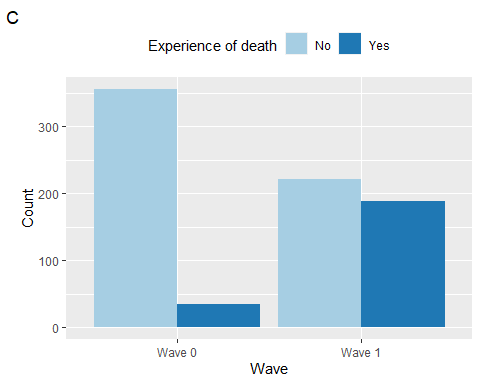


Sufficient PPE

df3 %>%
 filter(!is.na(PPE)) %>%
 ggplot(aes(x = wave, fill = PPE)) +
 geom_bar(position = "dodge") + labs(fill = "Sufficient PPE", x = "Wave", y = "Count", tag="D") +
 scale_fill_brewer(palette = "Paired") +
 theme(legend.position="top")


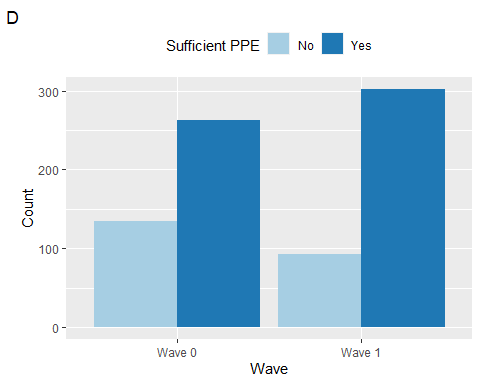

Supplement: Supplementary file 1 — Supplementary Information. [file 41598_2023_39735_MOESM1_ESM.docx]
